# Polar-Twisted Electron-Transport Layer Simultaneously Breaks Efficiency-Flexibility-Cost Limits in Organic Solar Cells

**DOI:** 10.1007/s40820-026-02330-5

**Published:** 2026-08-13

**Authors:** Thi Le Huyen Mai, Zhe Sun, Won Jung Kang, Seunglok Lee, Taek-Soo Kim, Changduk Yang

**Affiliations:** 1 Department of Energy Engineering, School of Energy and Chemical Engineering, Ulsan National Institute of Science and Technology (UNIST), 50 UNIST-gil, Ulju-gun, Ulsan, 44919 Republic of Korea; 2 Department of Mechanical Engineering, Korea Advanced Institute of Science and Technology (KAIST), 291 Daehak-ro, Yuseong-gu, Daejeon, 34141 Republic of Korea; 3 Graduate School of Carbon Neutrality, Ulsan National Institute of Science and Technology (UNIST), 50 UNIST-gil, Ulju-gun, Ulsan, 44919 Republic of Korea; 4 UNIST InnoCORE AI-Space Solar Initiative, Ulsan National Institute of Science and Technology (UNIST), Ulsan, 44919 Republic of Korea

**Keywords:** Electron-transport layers, Organic solar cell, Perylene diimide, Semitransparent flexible devices, Stability

## Abstract

Perylene diimide (PDI)-based electron-transport layers (ETLs) are fundamental in governing charge extraction, interfacial recombination, and the operational longevity of organic solar cells (OSCs), yet their molecular design still lacks transferable principles. Here we present a PDI-ETL molecular design framework that couples bay-position engineering with polar, bulky side-chain modulation to improve additive-free alcohol solubility, suppress over-crystallization, and mitigate oxidative degradation. With this framework, H75-DMA suppresses reactive-oxygen-driven chemical evolution and interfacial trap accumulation, thereby stabilizing interfacial energetics and electron transport. Binary OSCs based on H75-DMA achieve a power conversion efficiency (PCE) above 20% and exhibit improved mechanical toughness and bending durability in flexible devices. Its smooth, high-surface-energy interface also induces continuous growth of ultrathin metal cathodes, leading to increased light-utilization efficiency in flexible semitransparent OSCs. In addition, H75-DMA enables cathode substitution from Ag to Cu while retaining about 97% of the PCE of the Ag-based devices, thereby improving cost-effectiveness at the device level.

**Supplementary Information:**

The online version contains supplementary material available at 10.1007/s40820-026-02330-5.

## Highlights

- A polar-twisted perylene diimide electron-transport layers framework enables additive-free alcohol processing, suppressed over-crystallization, and improved oxidative stability.
- H75-DMA delivers over 20% efficiency in binary organic solar cells while retaining ~ 80% of its initial power conversion efficiency after 384 h air storage.
- H75-DMA enhances flexible and semitransparent organic solar cells and enables Ag-to-Cu cathode substitution with 18.65% efficiency, highlighting device-level cost effectiveness while approaching Ag-based performance.

**Supplementary Information:**

The online version contains supplementary material available at 10.1007/s40820-026-02330-5.

## Graphical abstract

**Figure:**
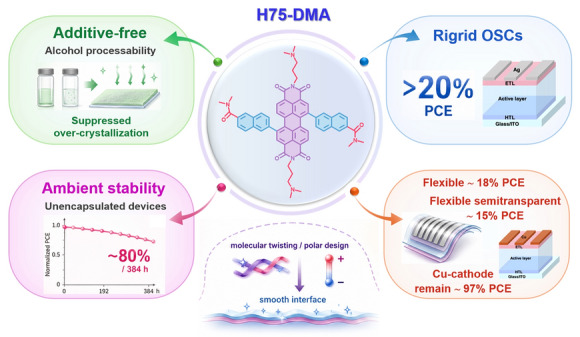

## Introduction

Perylene diimide (PDI) derivatives have emerged as a compelling molecular scaffold for electron-transport layers (ETLs) in organic solar cells (OSCs) owing to their excellent electron-transport characteristics and suitable energy-level alignment [1–12]. However, state-of-the-art PDI-based ETLs suffer from poor chemical stability, particularly under prolonged air exposure and illumination, leading to rapid performance degradation [13–19]. These reliability issues stem from two intertwined challenges. First, limited alcohol solubility is still a practical challenge for many neutral amine PDI ETLs, even though some well-known systems can be processed without acid additives [7, 11]. In such cases, processing aids such as AcOH are often introduced, which can complicate interfacial control and stability [17, 20–22]. Second, strong π–π stacking of planar PDI cores often yields excessive crystalline, edge-on films with rough surfaces that exacerbate interfacial recombination and mechanical brittleness. While crystallinity can facilitate charge transport [11, 23–26], excessive ordering undermines interfacial integrity and stability [16, 27–29].

**Fig. 1:**
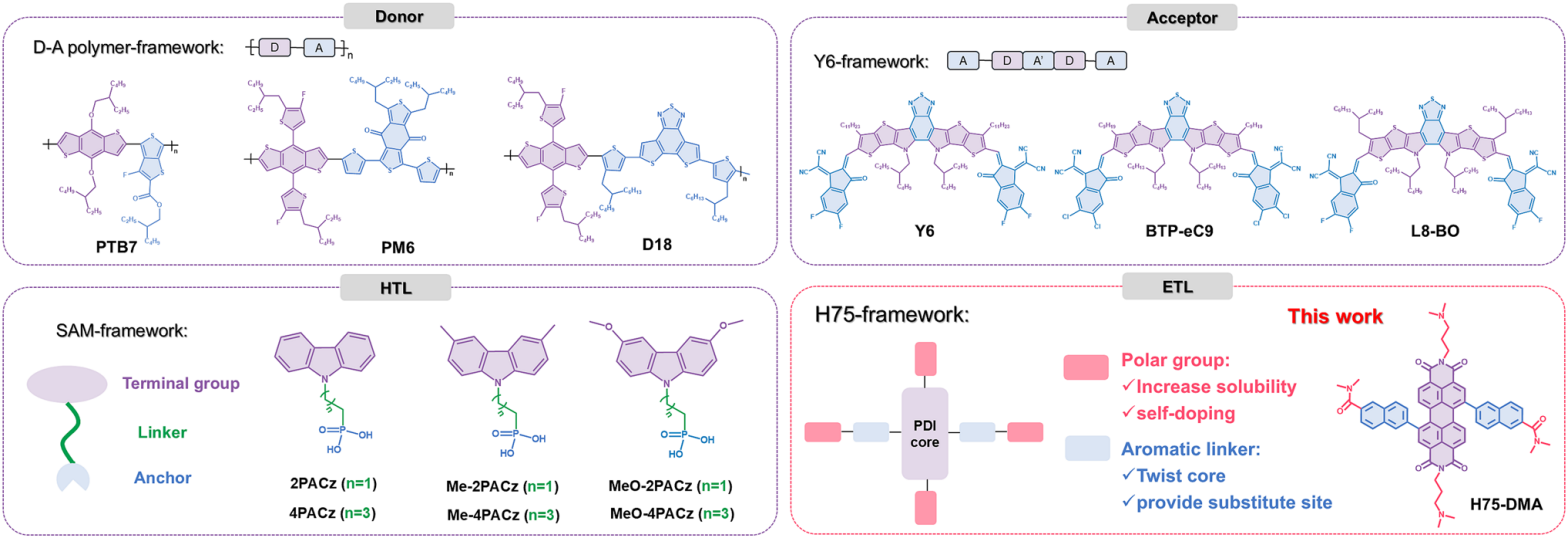
Molecular framework for OSC materials design

In the broader context of OSC development, the rapid advancement of other functional layers has been propelled by the establishment of molecular frameworks—such as donor—acceptor (D–A) polymer framework for donors, the Y6-series framework for acceptors, and self-assembled monolayer (SAM) frameworks for hole-transport layers (Fig. 1). These frameworks provide precise molecular design guidelines that have greatly accelerated the discovery of high-performance materials. By contrast, no comparable framework exists for PDI-based ETLs, despite their critical role in charge extraction and interface engineering. This absence of guiding principles for ETL design represents a major bottleneck in achieving simultaneous advances in efficiency, stability, and processability.

Here, we introduce an ETL molecular framework, termed the H75-framework that integrates sterically twisted bay substituents with polar functionalities to simultaneously modulate solubility, morphology, and oxidative stability. Using this strategy, we designed H75-DMA, which incorporates polar bulky dimethylnaphthylamide (DMA) units at the bay positions, and benchmarked it against PDIN-NAP with bay-position naphthyl groups and conventional PDIN and PDINN ETLs. The H75-DMA-based device achieves a power conversion efficiency (PCE) of 20.07%, retaining ~ 80% performance after 384 h of air storage, and enabled flexible OSCs with a crack-onset strain (COS) > 21% and 6.72 MJ m⁻^3^ toughness, maintaining 83% PCE after 2500 bending cycles. Moreover, H75-DMA supported excellent flexible semitransparent devices with a light-utilization efficiency ~ 3%, and enabled Ag-to-Cu-cathode substitution while still achieving 18.65% PCE, approaching Ag-based performance. Our studies define a generalized design strategy for PDI ETLs, exemplified by H75-DMA, that concurrently realizes high PCE, intrinsic stability, and improved device-level cost-effectiveness across diverse OSC architectures.

## Experimental Section

### Materials

All reagents and solvents for electron-transport material synthesis were purchased Aldrich, Alfa Aesar, Combi-Blocks, and used without any purification. Perylenetetracarboxylic dianhydride (PTCDA, 98%), 1-bromobutane, and p-toluenesulfonic acid monohydrate (p-TsOH·H_2_O), n-butanol, [1,1′-Bis(diphenylphosphino)ferrocene]dichloropalladium(II), 1,4-dioxane from Sigma-Aldrich. 1, Diazobicyclo[5.4.0]undec-7-ene (DBU), potassium carbonate (anhydrous 99%), acetic acid, dimethylamine, N,N-Dimethyl-1,3-propanediamine (99%), were purchased from Alfa Aesar. Bis(pinacolato)diboron, 6-bromo-2-naphthoic acid, naphthalene-2-boronic acid from Combi-Blocks. Bromine (99%), sodium bisulfate, magnesium sulfate, dichloromethane (DCM), acetonitrile (ACN), dimethylformamide (DMF), methanol, toluene, and triethylamine from Samchun chemical company. D18-Cl, PM6, L8-BO, and N3 were purchased from Derthon. Regarding the reference electron-transport materials, PDINN was purchased from SunaTech Inc., whereas PDIN and 1,7-dibromoperylene-3,4,9,10-tetracarboxylic acid dianhydride (PDIN-Br_2_) were synthesized according to previously reported procedures [16]. The detailed synthetic procedures and characterization data for the newly synthesized electron-transport materials are provided in the Supporting Information.

### Computational Methods

The structural geometries of ETL material were optimized at the B3LYP/6-31G(d,p) level of theory using Gaussian 09 software. And then, the files generated by Gaussian 09 were employed as input for Multiwfn software [30] to conduct the LBO analysis.

### Instruments and Characterizations

#### ^***1***^***H NMR, CV, UV–Vis, and Mass Spectrometry Measurement***

^1^H NMR spectra were collected on a Varian VNRS 400 MHz spectrometer at 298 K, using CDCl_3_ as the solvent. Cyclic voltammetry (CV) experiments were run on an AMETEK VersaSTAT 3 potentiostat in a standard three-electrode setup: glassy-carbon working electrode, platinum wire counter electrode, and Ag/Ag^+^ reference electrode. Measurements were performed at room temperature in nitrogen-purged acetonitrile containing 0.1 M n-Bu_4_NPF_6_, with a scan rate of 100 mV s^−1^. UV–visible absorption spectra were acquired on a Shimadzu UV-2550 spectrophotometer, and MALDI-TOF mass spectra were recorded with a Bruker Autoflex max instrument.

#### FTIR Measurement

The thick neat films of the ETL materials were drop-coated on the silicon substrates, and the samples were recorded with a Varian instrument.

#### XPS and UPS Measurement

The XPS and UPS data were recorded using a multifunctional photoelectronic energy spectrometer ESCALAB 250Xi (Thermo Fisher Scientific). For XPS measurements, a monochromated Al Kα X-ray source and a hemispherical energy analyzer were used. High-resolution spectra were collected at a pass energy of 30 eV with an energy step size of 0.1 eV, 4 scans, and a dwell time of 150 ms. Charge compensation was applied during the measurements. The binding-energy scale was calibrated using the C 1*s* signal of adventitious carbon at 284.8 eV. Samples were spin-coated onto glass substrates in a glovebox and then exposed to ambient conditions for the aging test. The XPS spectra were processed and fitted using CasaXPS software, and the detailed fitting parameters are summarized in Table S5.

#### Electron Spin Resonance (ESR) Measurement

The spectra were recorded on a Bruker EMXplus spectrometer operating in the X-band (9.3–9.9 GHz) at 300 K with a maximum microwave output of 200 mW, while microwave power and field modulation were carefully tuned to prevent signal saturation. The spin-trapping agent DMPO was used to probe both superoxide and hydroxyl radicals: For superoxide detection, ETL powder was dispersed for 30 min in a DMPO/methanol solution (1: 100 v/v), yielding the DMPO–OOH adduct, whereas hydroxyl radicals were monitored by dispersing ETL powder for 30 min in a DMPO/water solution (1: 100 v/v) to form the DMPO–OH adduct. Each dispersion was analyzed immediately after preparation and again after 168 h of storage under ambient conditions, with the aged samples displaying markedly stronger ESR signals than the fresh ones.

#### AFM, KPFM, and GIWAXS Measurement

Surface morphology was probed with a Veeco MultiMode V microscope equipped with a NanoScope controller, employing silicon tips from Bruker for AFM imaging and Pt/Ti-coated probes (SPARK 70 Pt, Nu Nano Ltd.) for surface-potential mapping by KPFM. Grazing-incidence wide-angle X-ray scattering (GIWAXS) measurements were performed at the Pohang Accelerator Laboratory (Korea) on the PLS-II 6D U-SAXS and 9A beamlines, where scattering patterns were captured with a Rayonix SX165 two-dimensional CCD detector. An X-ray energy of 11.24 keV was used, and the incidence angle was adjusted between 0.08° and 0.12° to maximize the signal-to-background ratio.

#### Device Characterization

The current density–voltage (*J–V*) measurements were measured on a Keithley 2400 source-measure unit with an intensity of 100 mW cm^–2^ under the illumination of an AM 1.5 G solar simulator. A standard Si solar cell was used to calibrate the light intensity of the solar simulator. The external quantum efficiency (EQE) measurements were conducted using Model QE-R3011 (Enli Technology) in ambient air. The aperture area of 0.0385 cm^2^ masks was used for testing devices.

#### TPV and TPC Measurement

The transient photovoltage (TPV) and transient photocurrent measurements (TPC) were conducted using the analyzer function of an organic semiconductor parameter test system (McScience T4000) under the illumination with a white light-emitting diode.

#### Electrochemical Impedance Spectroscopy (EIS) Measurement

The Nyquist plot, Bode plot, Mott–Schottky plot, and capacitance versus frequency/applied voltage curves were obtained by electrochemical workstation (Ivium, Netherlands).

#### Conductivity Measurement

ETLs in chloroform were spin-coated at a film thickness (d) of ~ 100 nm on substrates with parallel silver electrodes, and the conductivity of the ETLs was measured by the slope of the I–V plot using the equation I = σ × A × V × d^−1^, where d is the film thickness and A is the sample area.

#### Space-Charge-Limited Current (SCLC) Measurement

To assess electron-transport properties of the ETLs, electron mobility was quantified using the SCLC method in electron-only devices with the configuration ITO/ZnO/ETL/Ag. Dark *J–V* curves were recorded at room temperature under N₂ atmosphere. The electron mobility (μ) was extracted using the Mott–Gurney equation:

$$J=\frac{9}{8}{\varepsilon}_{0}{\varepsilon}_{r}\mu \frac{{V}^{2}}{{L}^{3}}$$

where ${\varepsilon}_{0}$ is the vacuum permittivity, ${\varepsilon}_{r}$ is the relative dielectric constant, μ is the electron mobility, V is the applied voltage, and L is the ETL thickness.

#### Stability Measurement

As it relates to the stability testing of the ETL materials, a sealed box containing a glass of water and a high humidity environment with a relative humidity (RH) of above 85% (~ 25 °C) was employed to ensure the RH value. Following that, each ETL sample was placed in a sealed box for 168 h.

Air and heat stability tests were performed on unencapsulated devices based on the D18-Cl:N3 system. For thermal stability testing, the cells were placed directly on a hot plate with an 85 °C temperature setting in a nitrogen-filled glove box. The PCE was occasionally measured after cooling to ambient temperature). In the air stability test, the cells were evaluated in the ambient environment (25 °C and 40%–60% RH), with room light as the light source.

Operational stability examinations of the OSC devices without encapsulation were performed utilizing a white light LED array under continuous illumination simulating an intensity of 100 mW cm^–2^ in an ambient environment (approximately 55 °C, 40%–60% RH), with J-V curves recorded over time (T3600, McScience). The device’s area of 0.0385 cm^2^ is utilized for stability measurements.

### Mechanical Properties Measurement

#### Pseudo-Freestanding Tensile Test

The pseudo-freestanding tensile method was carried out in accordance with the experimental protocols described in the previously published research [31]. The ETL was applied via spin-coating onto the glass/PSS/active layer substrate. Following the film deposition, the glass/PSS/active layer/ETL specimens were cut into a dog-bone configuration using a femtosecond laser, thereafter, transferred onto water by dissolving the underlying PSS sacrificial layer, and then attached to the grips using van der Waals forces. Tensile load values were determined using a load cell (LTS-50GA, KYOWA, Japan), whereas strain values were assessed by a digital image correlation (DIC) system. The strain was applied at a constant strain rate of 0.8 × 10^–3^ s^−1^. The elastic modulus was determined by employing the least squares fit to the linear segment of the stress–strain curve up to 1.0% strain. The crack-onset strain was identified as the strain at which the tensile load significantly decreased due to the commencement of cracking (Fig. S32).

#### Double Cantilever Beam (DCB) Test

Samples of the Si wafer/active layer/ETL were manufactured for the DCB test. The samples were cut into 8 mm × 40 mm strips and adhered on dummy silicon substrates with epoxy (353ND, EPO-TEK) to fabricate DCB specimens. The cohesive fracture values were subsequently determined utilizing a high-precision micromechanical testing equipment (Delaminator Adhesion Test System, DTS Company, Menlo Park, USA). The initial loading rate was set at 0.5 μm s^−1^. The final Gc value was determined by averaging the Gc values derived from each loading–unloading cycle. Interface analysis can be performed with techniques such as OM, AFM, and XPS; however, it was not conducted in this experiment as the interface was easy to identify to the human eye (Fig. S33).

$$\mathrm{C}\mathrm{r}\mathrm{a}\mathrm{c}\mathrm{k}~ \mathrm{l}\mathrm{e}\mathrm{n}\mathrm{g}\mathrm{t}\mathrm{h},a = {\left(\frac{C{E}^{^{\prime}}B{h}^{3}}{8}\right)}^\frac{1}{3}-0.64h$$

$${\mathrm{A}\mathrm{d}\mathrm{h}\mathrm{e}\mathrm{s}\mathrm{i}\mathrm{o}\mathrm{n}~ \mathrm{e}\mathrm{n}\mathrm{e}\mathrm{r}\mathrm{g}\mathrm{y},G}_{c} = \frac{12{{P}_{\mathrm{c}}}^{2}{a}^{2}}{{E}{^\prime}{B}^{2}{h}^{3}}{\left(1+\frac{0.64h}{a}\right)}^{2}$$

C = compliance

$${E}{^\prime} = Elastic~ modulus~ of ~beam \left(Si~ wafer\right)$$

B = width of beam

h = thickness of beam (half of total thickness)

$${P}_{\mathrm{c}} = Critical~ load$$

#### Bending Test

The bending test of flexible devices was conducted by ST-MS60-Bending (Sciencetown Company).

### AVT Value

$${\mathrm{AVT}} = \frac{\smallint T(\lambda )V(\lambda )S(\lambda )d(\lambda )}{{\smallint P(\lambda )S(\lambda )d(\lambda )}}$$

where λ denotes the wavelength, T represents the transmission, V signifies the normalized photopic spectral response of the eye, and S indicates the sun photon flux (AM 1.5 G). The average transparency of the devices in the visible spectrum (380–740 nm) is evaluated based on the photonic response of the human eye.

### Device Fabrication

The organic solar cell devices (OSCs) were constructed using the conventional architecture of ITO/PEDOT:PSS/active layer/electron-transport layer (ETL)/Ag. The ITO glasses were sequentially cleaned with detergent, deionized water, acetone, and isopropyl alcohol using a sonication device, and subsequently dried overnight in an oven. Following a 20-min UV-Ozone treatment, PEDOT:PSS (Bayer Baytron 4083) was spin-coated onto ITO glasses at 4000 rpm, resulting in a thickness of approximately 30 nm, and subsequently annealed at 150 °C for 20 min in ambient air. The blend solution of the D18-Cl: N3 system, particularly D18-Cl:N3 (1.0: 1.4) in CF at a concentration of 10 mg mL^−1^, was spin-coated at 2200 rpm and subsequently annealed at 80 °C for 10 min, with DIB added at a mass fraction of 60%. In case of PM6:L8-BO system, the blend solution of PM6:L8-BO (1.0: 1.2) in CF at 16 mg mL^−1^ was spin-coated at 2000 rpm before annealing at 100 °C for 10 min. Subsequently, a methanol solution of PDIN (2.0 mg mL^−1^, 0.3% acetic acid), PDIN-NAP (2.0 mg mL^−1^, 0.1% acetic acid), H75-DMA (2.0 mg mL^−1^), or PDINN (1.0 mg mL^−1^) was spin-coated onto the active layer at 3000 rpm for 30 s. Finally, silver cathode (~ 100 nm) was thermally deposited onto the substrates in a high vacuum environment (< 3.0 × 10^–6^ Pa).

The flexible organic solar cells were constructed using PET/ITO/PEDOT:PSS/D18-Cl:N3/ETL/Ag. The flexible electrode (PET/ITO) was attached to glass substrates using tape, followed by the normal spin-coating procedure for the device. The PEDOT:PSS solution was subsequently spin-coated and annealed on a hot stage at 100 °C for 15 min. Next, the process conditions match those of the rigid device.

The semitransparent flexible organic solar cells (OSCs) with the configuration of PET/ITO/PEDOT:PSS/D18-Cl:N3/ETL/Ag/MoO_3_ were fabricated according to the specified process conditions for flexible OSCs. The thickness of the Ag electrode was 20 nm. Finally, 35 nm of MoO_3_ was thermally vaporized at a rate of 0.3 Å s^−1^ to form the optical structure.

## Results and Discussion

### Design, Properties, and Photovoltaic Performance of ETLs

**Fig. 2:**
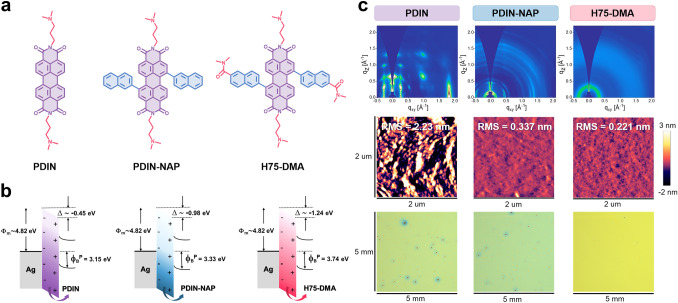
Material properties of ETLs. **a** Chemical structures of PDIN, PDIN-NAP, and H75-DMA. **b** Schematic energy-level diagrams illustrating how each ETL lowers the Ag work function via interfacial dipole formation. **c** Film morphology of the ETLs: 2D GIWAXS patterns (top), AFM height images (middle), and optical-microscopy images (bottom)

To explore the role of H75-framework as the ETL in OSCs, we synthesized PDIN-NAP and H75-DMA via Suzuki–Miyaura coupling of PDIN-Br_2_ with naphthyl (NAP) and DMA pinacol boronic esters, respectively (Figs. 2a and S1). Their structures and purity were confirmed by ^1^H and ^13^C NMR spectroscopy and MALDI-TOF mass spectrometry (Figs. S2–S7, S45, and S46). Density functional theory geometry optimizations at B3LYP/6-31G* revealed that the NAP units at the bay positions alleviate the planarity of PDI backbone, introducing a twist angle over ~ 19°, which likely leads to reduced intermolecular aggregation (Fig. S8). Notably, H75-DMA can be readily deposited as an ETL from alcohol-based solvents without any acidic additive, whereas AcOH is required for PDIN (0.3 vol%) and PDIN-NAP (0.1 vol%) to form suitable ETL films. These results indicate that both the planarity-broken backbone and polar DMA units at bay-position serve as the primary factor governing alcohol-solvent processibility of the H75-framework. PDIN-NAP and H75-DMA show similar absorption features in both methanol solution and film, with redshifted and broadened spectra relative to PDIN because of the extended bay-position conjugation. Both PDIN-NAP and H75-DMA have higher-lying highest occupied molecular orbitals (HOMOs) and deeper-lying lowest unoccupied molecular orbitals (LUMOs) than PDIN, as determined by cyclic voltammetry. Their frontier energy levels align well with those of commonly used acceptors in OSCs, enabling efficient hole blocking and electron collection. The relevant optical and electrochemical properties are summarized in Figs. S9, S10 and Table S1.

Ultraviolet photoelectron spectroscopy (UPS) shows that H75-DMA-deposition more effectively reduces the Ag work function to 3.58 eV, compared to 3.84 eV for PDIN-NAP and 4.37 eV for PDIN (Figs. 2b, S11 and Table S2). This results in a higher hole-injection barrier (3.74 eV) for H75-DMA, thereby suppressing interfacial recombination and enhancing *V*_OC_ [32]. Electron spin resonance (ESR) further indicates that H75-DMA produces the strongest radical signal, reflecting its superior intrinsic self-doping capability relative to PDIN-NAP and PDIN (Fig. S12) [32,33]. These results correlate well with the enhanced charge mobility and conductivity observed in H75-DMA films compared with PDIN-NAP and PDIN (Figs. S13, S14 and Table S3).

As shown in Fig. 2c, grazing-incidence wide-angle X-ray scattering (GIWAXS) patterns of PDIN exhibits clear (100) out-of-plane and (010) in-plane peaks, along with numerous diffraction spots, indicating a preferentially edge-on oriented polycrystalline structure. These distinct diffraction patterns became progressively weaker in PDIN-NAP and were virtually absent in H75-DMA, signifying the loss of long-range molecular ordering and crystallinity. Atomic force microscopy (AFM) height images confirm that H75-DMA yields the smoothest surface, with an RMS roughness of 0.221 nm, compared with 0.337 nm for PDIN-NAP and 2.23 nm for PDIN (Fig. S15). Consistently, optical microscopy reveals a uniform, featureless surface for H75-DMA, whereas particulates are visible in PDIN-NAP and are more pronounced in PDIN (Fig. 2c). This amorphous-like morphology gives H75-DMA a more homogeneous interface, which is expected to favor charge extraction and suppress interfacial recombination (Fig. S16).

**Fig. 3:**
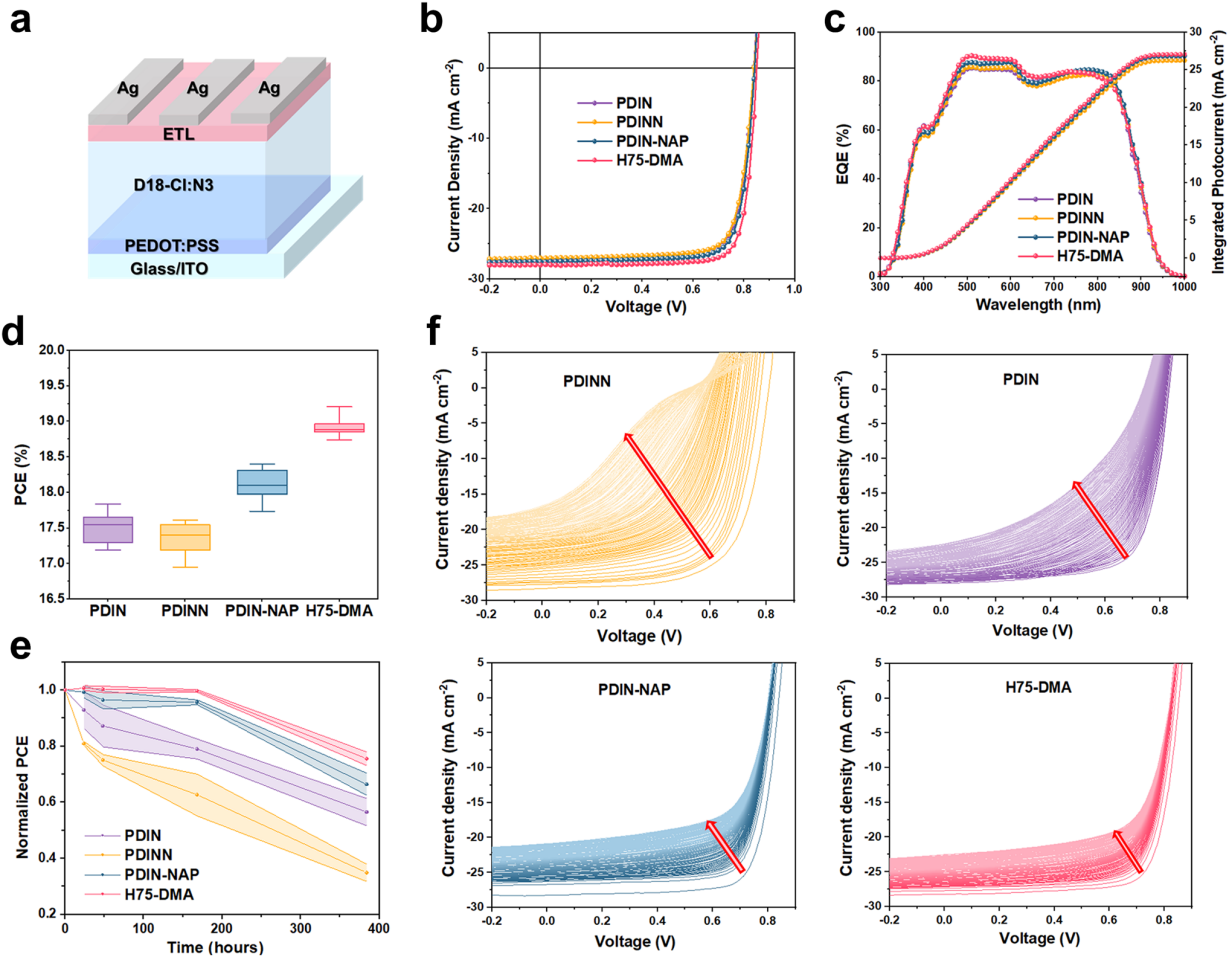
Photovoltaic performance and stability of optimized OSCs. **a** Device architecture for performance optimization. **b** *J*–*V* curves of the optimized devices with various ETLs under illumination of AM1.5 G, 100 mW cm^−2^. **c** EQE spectra and integrated current densities. **d** Statistical distribution of initial PCEs. **e** Normalized PCEs of unencapsulated devices stored under ambient conditions (~ 25 °C, 40%–60% RH). **f** *J* − *V* curves recorded during accelerated light-aging under continuous 1-sun illumination at 55 °C and 40%–60% RH using an automated *J* − *V* tracker without an aperture mask. The panel is intended to show the evolution of* J*–*V* curve shape during aging

OSC devices with the architecture ITO/PEDOT:PSS/D18-Cl:N3/ETL/Ag were fabricated (Fig. 3a). For the following performance and stability studies, we also included PDINN—a highly soluble PDIN analog containing additional secondary amine (–NH–) groups in its side chains—as a reference ETL to better examine the structure–property–stability relationship (Figs. 2a and S17). Notably, PDINN can be processed from alcohol solvents without AcOH, like H75-DMA, and thus serves as an acid-free reference to minimize the influence of AcOH in the stability comparisons. The current density–voltage (*J*–*V*) curves and external quantum efficiency (EQE) spectra of the representative devices are presented in Fig. 3b, c, and the corresponding photovoltaic parameters are summarized in Table 1. The H75-DMA-based device showed the highest PCE of 19.21%, with a *V*_OC_ of 0.847 V, a short-circuit current (*J*_SC_) of 27.96 mA cm^−2^, and a fill factor (FF) of 81.01%. In comparison, devices based on PDIN-NAP, PDIN, and PDINN show lower PCEs of 17.70–18.25%. The statistical distribution of 16 independent devices further confirms the performance advantage of H75-DMA (Fig. 3d). As shown in Fig. S18, recombination losses were evaluated from the light intensity (*I*) dependence of *J*_SC_ and *V*_OC_, using the relationships *J*_SC_ ∝ *I*^α^ and the slope (*kT*/*q*) in the ln(*I*)–*V*_OC_ plot. Compared with the other ones, the H75-DMA-based device shows a higher *α* of 0.999 and a smaller of 1.178 *kT*/*q*, suggesting lower biomolecular and trap-assisted recombination losses [34–37]. To further validate the potential of H75-DMA in high-performance OSCs, we employed 2PACz as HTL and achieved a PCE of 20.07% in the PM6:L8-BO binary system (Fig. S19 and Table 1).

**Table 1:** Optimized photovoltaic parameters of devices based on PDIN, PDIN-NAP, and H75-DMA as the ETL under the illumination of AM 1.5G, 100 mW cm^−2^

Active layer	ETLs	*V*_OC_^a^ [V]	*J*_SC_^a^ [mA cm^−2^]	*J*_cal_ [mA cm^−2^]	FF ^a^ [%]	PCE ^a^ [%]
D18-Cl:N3	PDIN	0.837 (0.835 ± 0.003)	27.15 (27.14 ± 0.09)	26.28	77.88 (77.10 ± 0.61)	17.70 (17.49 ± 0.19)
	PDINN	0.837 (0.835 ± 0.003)	27.03 (27.03 ± 0.11)	26.24	77.64 (76.92 ± 0.74)	17.58 (17.36 ± 0.19)
	PDIN-NAP	0.840 (0.840 ± 0.002)	27.52 (27.49 ± 0.23)	26.84	78.90 (78.39 ± 0.40)	18.25 (18.11 ± 0.19)
	H75-DMA	0.847 (0.846 ± 0.001)	27.96 (27.90 ± 0.07)	27.05	81.01 (80.16 ± 0.29)	19.21 (18.91 ± 0.12)
PM6:L8-BO	H75-DMA	0.893 (0.846 ± 0.009)	27.57 (27.59 ± 0.15)	26.47	81.48 (80.10 ± 0.51)	20.07 (19.54 ± 0.27)

^a^The average PCE values are obtained from 16 cells

Unencapsulated stability tests in air further highlight the advantage of H75-DMA. After 384 h of ambient storage (~ 25 °C, 40–60% RH), H75-DMA-based devices retained approximately 80% of its initial PCE, whereas PDIN-NAP-based device maintained ~ 70%. In contrast, the PDIN- and PDINN-based devices suffered pronounced degradation, with PCE losses exceeding 40% and 50%, respectively (Figs. 3e, S20 and Table S4,). Under continuous one-sun illumination at heating 55 °C under 40%–60% RH, the H75-DMA-based device displayed only minor changes in *J*_SC_, *V*_OC_, and FF, whereas the others showed progressively greater losses across three metrics (Figs. 3f and S21). A similar trend was observed in dark thermal aging under N_2_ (Fig. S22).

### Mechanistic Insights into Oxidative Degradation and Interfacial Stability

To elucidate the oxidative degradation pathway of the ETLs, we performed Laplacian bond order (LBO) calculations to assess their bond stability (Fig. S23) [38]. Compared to the LBOs (0.701–0.719) observed for the side chains of the other ETLs, the PDINN had lower LBO values (0.478–0.694) associated with the secondary amine (N–H) and the neighboring C–H bonds at positions 3 and 4, as shown in Fig. S24. This suggests a high propensity for hydrogen abstraction by reactive-oxygen species (ROS), thereby rationalizing the accelerated degradation observed for PDINN. Notably, for PDIN subjected to AcOH treatment, the LBO values of all bonds within the side chains were reduced (Fig. S25). In a comparison between ETLs with and without bay-position functionalities, the LBO values of the C–H bonds adjacent to the PDI core were found to be similar. Note that in the cases of PDIN-NAP and H75-DMA, the $\mathrm{C}_{sp^{2}}$–$\mathrm{C}_{sp^{2}}$ bonds formed at the bay 1,7-positions of PDI are slightly higher LBOs than the corresponding C–H bonds (Fig. S26).

To probe the actual stability of the ETLs, we performed a series of structural characterizations before and after one week (168 h) of storage in air. First, we tracked their NMR spectra using MeOH-*d*_4_ (2 mg mL^−1^) as the solvent (Figs. S27 and S41–S44). All the ETLs showed characteristic aromatic protons of PDI core in the range of 8–6 ppm with imide side-chain protons appearing between 4.5 and 1.5 ppm. After one week of storage in air, both H75-DMA and PDIN-NAP exhibited identical NMR resonance peaks, while PDIN and PDINN showed significant changes in peak intensity, broadening, and multiplicity, suggesting substantial structural and/or chemical alterations. These findings validate that the incorporation of bay-positioned NAP groups plays a critical role in enhancing the intrinsic stability of PDI-based ETLs.

**Fig. 4:**
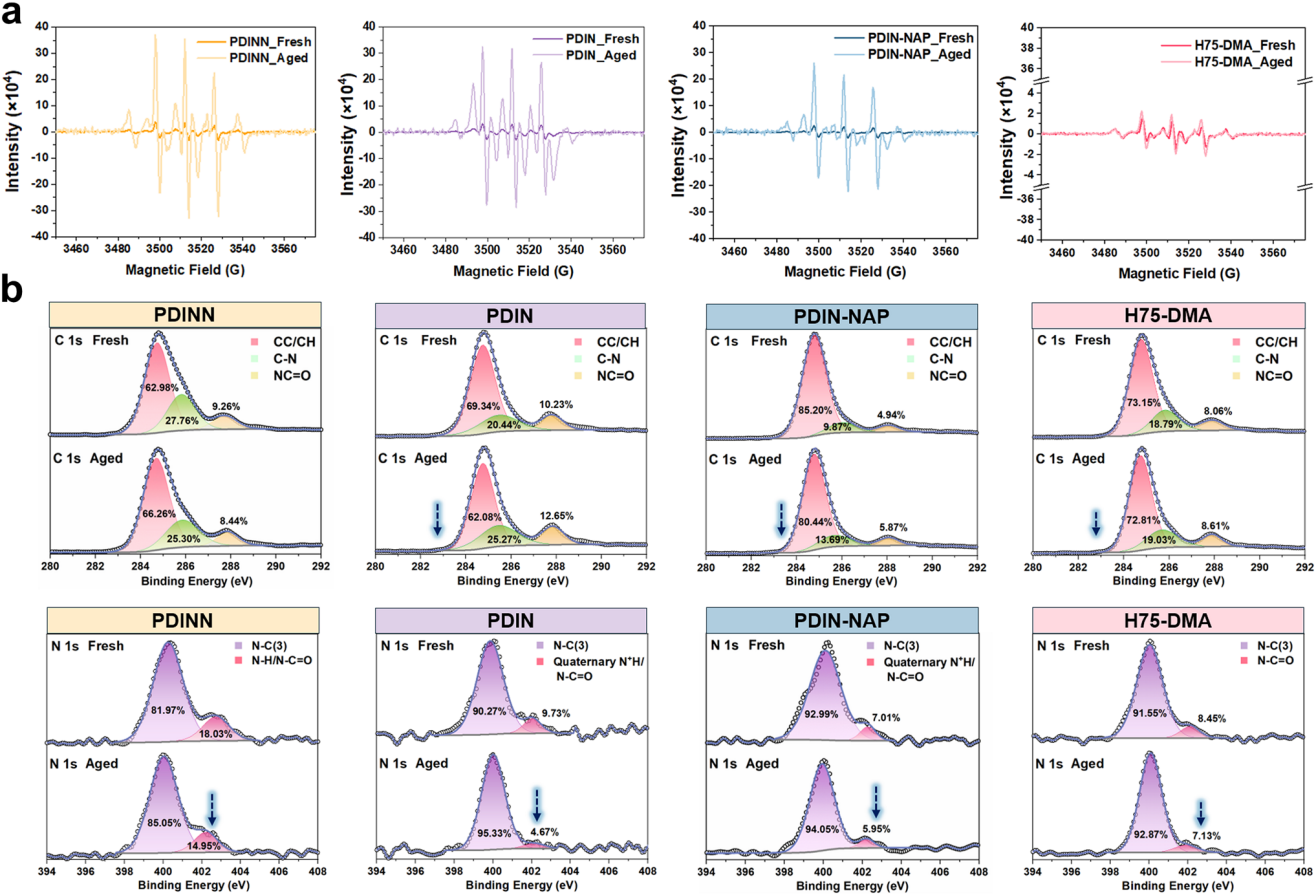
Ambient stability of ETLs (before and after one week (168 h) at 25 °C and 40%-60% relative humidity). **a** ESR signals of O_2_^−^ (DMPO–^•^OOH) for fresh and aged samples of PDIN, PDINN, PDIN-NAP, and H75-DMA in methanol solution. **b** XPS profiles of C 1*s* and N 1*s* from fresh and aged PDIN, PDINN, PDIN-NAP, and H75-DMA films

ESR spectroscopy with the spin-trapping agent (5,5-dimethyl-1-pyrroline *N*-oxide (DMPO)) offers a sensitive probe for evaluating chemical stability by directly detecting superoxide and hydroxyl radicals in ETL solutions [39–41]. All the fresh samples displayed the characteristic ESR signals of the DMPO–^•^OOH and DMPO–^•^OH adducts (Figs. 4a and S28). Following one week of storage in air, the DMPO adduct signals increased substantially for PDINN, consistent with the high oxidative reactivity of the secondary amine (N–H) moieties in its side chains. PDIN and PDIN-NAP also showed clear increases in adduct intensity, which we attribute to the involvement of AcOH-induced protonation (R_3_NH^+^) and related side reactions during processing. In contrast, H75-DMA exhibited only minor changes, supporting its superior resistance to oxidative degradation.

We also compared the Fourier-transform infrared (FTIR) spectra of the ETLs before and after storage in air (Fig. S29). We identified the characteristic absorption bands corresponding to distinct functional groups in ETL films, including N–H stretching at ~ 3300 cm^−1^, C–H aliphatic and aromatic stretching at 2800–3000 cm^−1^, C=O imide stretching at ~ 1700 cm^−1^, and C=C aromatic stretching at ~ 1650 and 1600 cm^−1^. Upon closer examination, it was found that after air storage, the peak intensities of PDIN and PDINN decreased, whereas those of PDIN-NAP and, in particular, H75-DMA remained largely unchanged, consistent with the ESR results discussed above. The negligible spectral changes for H75-DMA confirm their excellent resistance to the chemical transformations that plague the benchmark interlayers.

The surface changes and degradation can be seen in more detail from the X-ray photoelectron spectroscopy (XPS) data of the ETL films (Fig. 4b; fitting details are provided in Table S5). The C 1*s* spectra showed peaks at ~ 285 (C–C/C–H), ~ 286 (C–N), and ~ 288 eV (N–C=O), while the N 1*s* spectra exhibited signals at ~ 400 eV (N–C) and a higher-binding-energy component near ~ 402 eV, corresponding to nitrogen-containing species in different chemical environments. After air storage, PDIN and especially PDINN showed clear redistribution of the C 1*s* and N 1*s* components, whereas PDIN-NAP changed more moderately and H75-DMA showed only minor variation. Taken together, the much smaller spectral changes of H75-DMA confirm its superior resistance to air-induced chemical degradation, in good agreement with the ESR and FTIR results.

As illustrated in Fig. S30, photooxidation is, in principle, a light-induced chemical process in which UV or visible photons excite organic materials used in organic solar cells (OSCs), enabling their interaction with molecular oxygen (O_2_) to generate various ROS such as singlet oxygen (^1^O_2_), superoxide (O_2_^–•^), or hydroxyl radicals (•OH) [42–48]. In OSC architectures, organic ETLs are particularly prone to photoexcitation-induced degradation, with specifically vulnerable hydrogen-containing sites such as quaternary N⁺H, secondary amine N–H, and C–H bonds adjacent to nitrogen atoms (excluding those in imide unit) located within the side chains. The air stability trends of the ETLs observed above demonstrate a clear dependence on the content of vulnerable hydrogen sites, among which the secondary amine N–H proves to be the most susceptible to degradation [49].

**Fig. 5:**
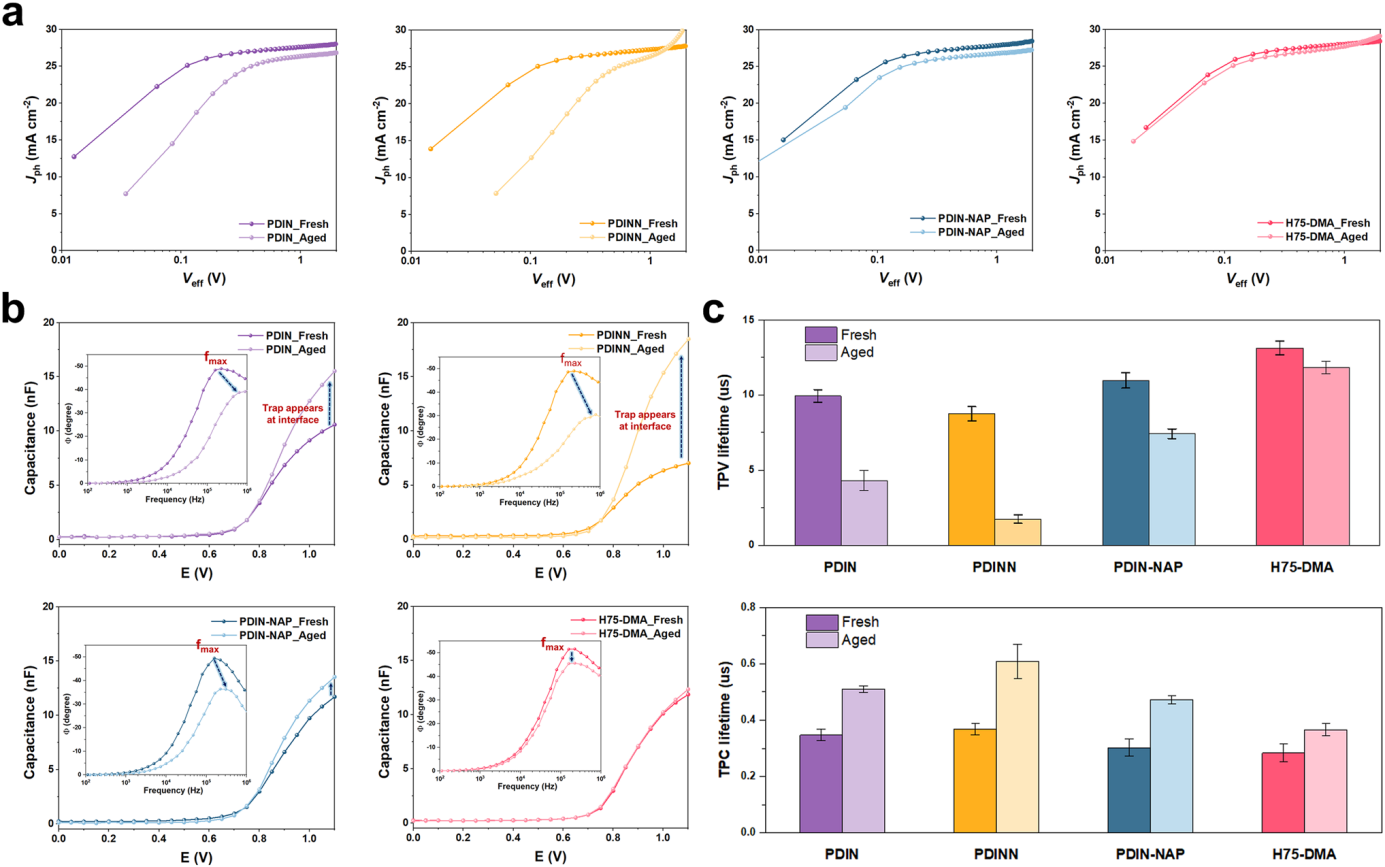
Charge dynamics analysis of fresh and aged OSCs before and after one week (168 h) at 25 °C and 40%–60% relative humidity. **a** *J*_ph_–*V*_eff_ curves of devices with different ETLs. **b** Capacitance versus applied voltage with corresponding Bode plots. **c** TPV and TPC lifetimes of fresh and aged devices

Figure 5a (see Table S6 for detailed values) presents the photocurrent density-effective voltage (*J*_ph_–*V*_eff_) profiles of fresh devices and those stored in air. For the H75-DMA-based devices, both exciton-dissociation (η_diss_) and charge-collection (η_coll_) efficiencies remain virtually unchanged after air exposure, in sharp contrast to the pronounced losses seen in PDIN-NAP-, PDIN-, and PDINN-based devices. Frequency-resolved capacitance–voltage (*C*–*V*) measurements (Fig. 5b) can serve to elucidate impact of aging on the interfacial trap landscape [50]. After air exposure, PDIN- and PDINN-based devices showed pronounced ΔC values (~ 5 and ~ 9 nF, respectively) along with a marked downward shift in the Bode phase peak, indicating substantial trap generation and charge accumulation at interface. The PDIN-NAP-based device exhibited a moderate ΔC (~ 2 nF), while the H75-DMA-based device showed minimal change (< 1 nF), indicating a well-preserved, trap-free interface. Impedance spectroscopy (Fig. S31) further corroborates these findings: Aged PDIN-NAP-, PDIN-, and PDINN-based devices possessed higher electron-transport resistance (R_tr_) and lower recombination resistance (R_rec_) relative to H75-DMA-based device [51]. These results demonstrate the critical role of the H75-DMA in not only suppressing interfacial traps and resistive barriers but also maintaining efficient charge transport under air exposure.

Transient photocurrent (TPC) and transient photovoltage (TPV) measurements (Fig. 5c, Table S7) were used to compare charge-extraction kinetics and carrier lifetimes in devices before and after air exposure [52–54]. In fresh devices, H75-DMA exhibited the fastest TPC decay and the longest TPV lifetime, followed by PDIN-NAP, PDIN, and PDINN, in accordance with the trend in initial PCE trend. Upon air aging, PDIN- and PDINN-based devices showed pronounced delays in TPC decay and reductions in TPV lifetime, in stark contrast to the stable performance of the H75-DMA-based device. These data correlate well with the interfacial trap formation and charge transport characteristics discussed above.

### Versatile Application Studies of ETLs

**Fig. 6:**
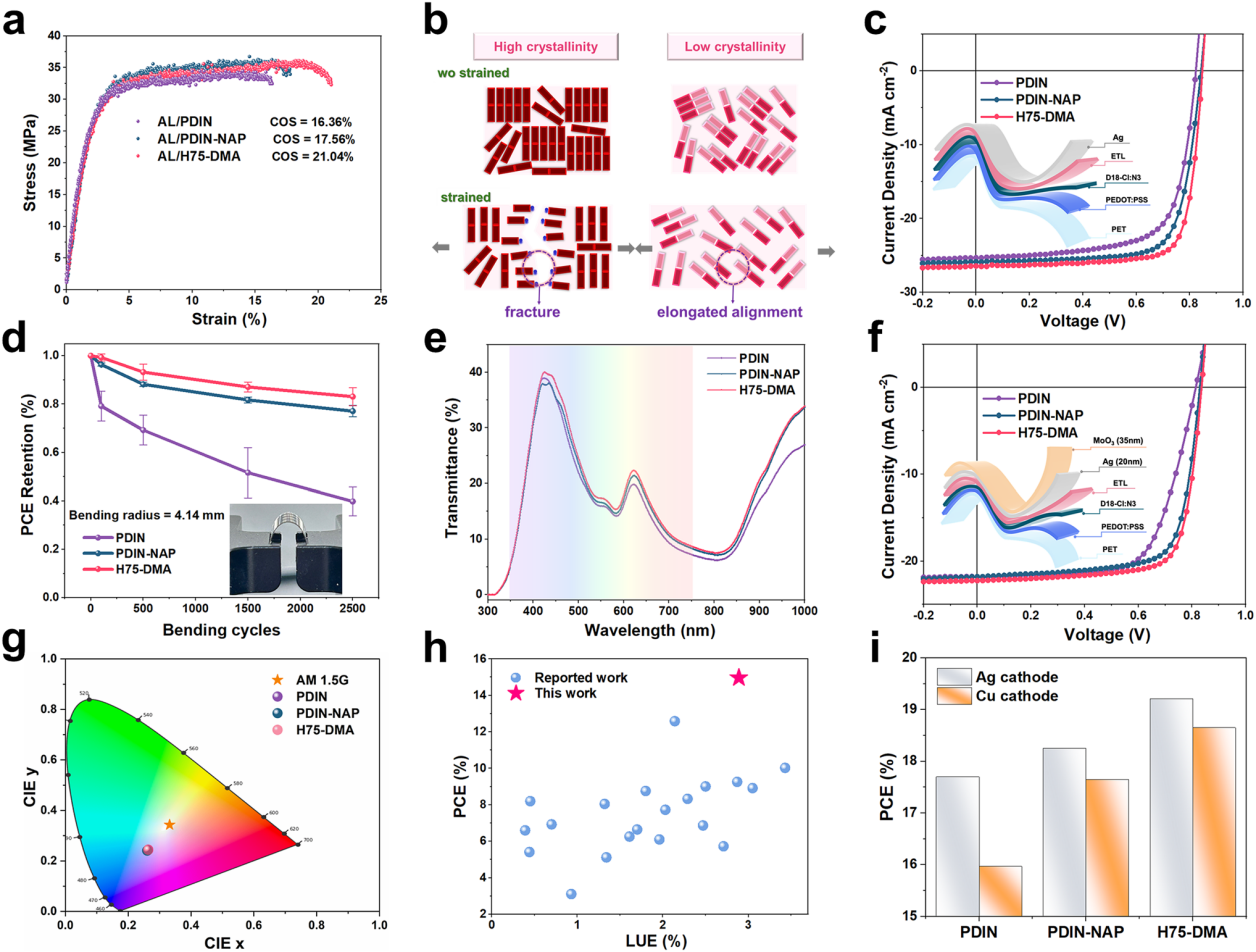
Versatile applications of ETLs across diverse OSC architectures. **a** Stress–strain curves of AL/PDIN, AL/PDIN-NAP, and AL/H75-DMA films from pseudo-freestanding tensile tests. **b** Schematic illustration of strain-induced deformation in crystalline versus low-crystallinity ETL films. **c** *J–V* curves of the flexible OSCs with different ETLs. **d** PCE retention for flexible OSCs as a function of bending cycles (radius = 4.14 mm). **e** Transmittance spectra of the semitransparent flexible OSCs with different ETLs. **f** *J*–*V* curves of the representative semitransparent flexible OSCs. **g** CIE 1931 chromaticity coordinates of the semitransparent flexible OSCs. **h** PCE and LUE for comparative literature data of semitransparent flexible OSCs (Supplementary Table 10). **i** PCE of OSCs employing Ag versus Cu cathodes with various ETLs

As shown in Fig. 6a, stress–strain curves of the active layer films incorporating different ETLs were obtained using a pseudo-freestanding tensile testing method to evaluate their tensile properties (Fig. S32) [31]. The H75-DMA-based film showed a higher COS exceeding 21%, yielding the highest toughness of 6.72 MJ m^–3^, compared with 16.4%–17.6% and toughness values of 5.00–5.69 MJ m⁻^3^ for the other films, as shown in the optical-microscopy images acquired during stress–strain testing. The detailed data are summarized in Figs. S33 and S34. Double-cantilever beam tests (Fig. S35) [55, 56] revealed a cohesive fracture energy (G_c_) of 4.39 J m^–2^ for H75-DMA, markedly exceeding those of PDIN-NAP (3.42 J m^–2^) and PDIN (3.32 J m^–2^), reinforcing the conclusion that H75-DMA-based film exhibits exceptional mechanical durability. Taken together with the tensile-stretching behavior, these results suggest that amorphous-like and less-ordered molecular domains accommodate deformation, enabling higher strain tolerance, as schematically illustrated in Fig. 6b.

To verify the advantages of H75-DMA in a flexible device setting, flexible OSCs were fabricated using the same device architecture described above, except with PET substrates instead of ITO. Figure 6c displays the *J*–*V* curves. Figures S36 and S37 present the PCE histograms, statistical distributions, and detailed values of each photovoltaic parameter, all of which are summarized in Table 2. The H75-DMA-based flexible device exhibited a higher PCE of 17.89% (*V*_OC_ of 0.845 V, *J*_SC_ of 27.21 mA cm^−2^, and FF of 77.81%) than those of the PDIN-based (15.40%) and PDIN-NAP-based (17.08%) counterparts.

**Table 2:** Optimized photovoltaic parameters of flexible devices based on PDIN, PDIN-NAP, and H75-DMA as the ETL on D18-Cl:N3 system under the illumination of AM 1.5G, 100 mW cm^−2^

Types of OSC devices	ETLs	*V*_OC_^d^ [V]	*J*_SC_^d^ [mA cm^−2^]	FF ^d^ [%]	PCE ^d^ [%]	AVT [%]	LUE [%]
Flexible devices^a^	PDIN	0.821 (0.825 ± 0.009)	26.85 (26.61 ± 0.29)	69.84 (67.62 ± 2.01)	15.40 (14.86 ± 0.43)		
	PDIN-NAP	0.839 (0.834 ± 0.007)	27.11 (26.92 ± 0.33)	75.07 (74.73 ± 0.55)	17.08 (16.79 ± 0.25)		
	H75-DMA	0.845 (0.839 ± 0.007)	27.21 (27.04 ± 0.28)	77.81 (76.66 ± 0.59)	17.89 (17.41 ± 0.29)		
Semitransparent devices^b^	PDIN	0.813 (0.809 ± 0.01)	23.12 (23.17 ± 0.21)	66.74 (63.29 ± 2.28)	12.56 (11.88 ± 0.51)	17.61	2.21
	PDIN-NAP	0.827 (0.827 ± 0.002)	23.47 (23.39 ± 0.15)	73.03 (72.69 ± 0.96)	14.19 (14.07 ± 0.19)	18.44	2.62
	H75-DMA	0.834 (0.834 ± 0.001)	23.95 (23.77 ± 0.26)	74.94 (74.45 ± 0.38)	14.97 (14.78 ± 0.16)	19.28	2.89
Cu-cathode devices^c^	PDIN	0.803 (0.805 ± 0.004)	27.15 (27.26 ± 0.24)	71.73 (71.71 ± 0.48)	15.63 (15.74 ± 0.20)		
	PDIN-NAP	0.833 (0.833 ± 0.001)	27.64 (27.48 ± 0.18)	76.62 (76.32 ± 0.76)	17.65 (17.48 ± 0.11)		
	H75-DMA	0.842 (0.841 ± 0.001)	27.85 (27.59 ± 0.22)	79.46 (79.18 ± 0.16)	18.65 (18.38 ± 0.19)		

^a^The flexible devices with the structure: PET/ITO/PEDOT:PSS/D18-Cl:N3/ETL/Ag (100 nm). ^b^The flexible semitransparent devices with the structure: PET/ITO/PEDOT:/PSS/D18-Cl:N3/Ag (20 nm)/MoO3 (35 nm). ^c^The Cu-cathode devices with the structure: Glass/ITO/PEDOT:PSS/D18-Cl:N3/Cu (100 nm). ^d^The average PCE values are obtained from at least 8 cells

We then evaluated the mechanical stability by subjecting the devices to repeated bending cycles with a radius of 4.14 mm (Fig. 6d and Table S8). The PDIN-based flexible device experienced severe burn-in mechanical loss, with PCEs rapidly declining by over 60% after 2,500 bending cycles. In sharp contrast, the PDIN-NAP and H75-DMA-based counterparts demonstrated substantially improved bending stability, with H75-DMA in particular retaining 83% of its initial PCE under identical conditions.

In semitransparent OSCs, ultrathin Ag cathode was evaporated onto the ETL, and its nucleation and growth behavior (Fig. S38a) were dictated by the underlying ETL surface. This in turn determines whether the Ag film grows continuously or as isolated islands, directly affecting its conductivity and transparency [57]. To directly quantify the ETL surface wettability that governs Ag nucleation/coalescence, we further measured three-liquid contact angles (DI water/ethylene glycol/diiodomethane) on the ETL surfaces and calculated the surface-energy components using the Owens–Wendt method (Fig. S39 and Table S9). Given the higher surface energy and smoother, more uniform surface of H75-DMA (Fig. 2c), we evaluated its templating ability by depositing ~ 20 nm Ag on PDIN, PDIN-NAP, and H75-DMA films and measuring the resulting sheet resistance and transmittance (Figs. 6e and S38b). Ag on PDIN showed high resistance and low transmittance; PDIN-NAP gave only slight gains; Ag on H75-DMA, however, achieved the lowest resistance and highest transmittance, evidencing a continuous film. We also fabricated flexible semitransparent OSCs (see inset of Fig. 6f). All the devices had similar chromatic coordinates located close to the AM 1.5 G reference point in CIE 1931 chromaticity diagram (Fig. 6g), indicating their excellent color-rendering performance. Relative to the other counterpart devices, H75-DMA-based flexible semitransparent device showed the highest LUE (PCE × AVT) around 3%, enabled by its superior PCE and average visible transmission (AVT). This LUE value is among the leading results reported for flexible semitransparent OSCs, with a PCE of ~ 15% (Figs. 6h, S10 and Table 2).

To demonstrate device-level cost reduction through cathode replacement, we further fabricated OSCs using a higher-work-function Cu cathode in place of Ag, noting that Ag is approximately 100 times more expensive than Cu by weight. Again, among the Cu-deposited low-cost devices, the H75-DMA-based device achieved the highest PCE of 18.65% (Figs. 6i, S40 and Table 2), approaching that of the Ag-deposited counterpart demonstrated earlier. Collectively, these diverse application studies confirm the broad advantages of H75-DMA for various types of OSC architectures.

## Conclusions

In summary, we proposed a unified PDI-based ETL framework through comparative investigations of benchmark PDI ETLs and newly designed derivatives (PDIN-NAP and H75-DMA) incorporating NAP and polar DMA units at the bay positions. Notably, H75-DMA afforded methanol solubility without acidic additives, formed uniform low-crystallinity films, and showed exceptional oxidative stability by reducing vulnerable C–H/N–H bonds and disrupting π–π aggregation, thereby suppressing key degradation pathways. H75-DMA-based device delivered a high PCE of 20.07% and enhanced long-term operational stability, retaining ~ 80% of initial PCE after 384 h of air storage. In flexible devices, H75-DMA film exhibited a COS exceeding 21% and toughness of 6.72 MJ m⁻^3^, delivering 17.89% PCE with nearly 83% retention after 2500 bending cycles. In flexible semitransparent devices, the synergy of high transmittance and low sheet resistance yielded a record-high LUE around 3%, among the highest reported for flexible semitransparent OSCs. Moreover, replacing Ag with a Cu cathode still enabled a competitive PCE of up to 18.65%, approaching that of the Ag-based counterpart and demonstrating improved cost-effectiveness at the device level. This unified molecular framework provides a conceptual foundation for designing high-performance, stable, and scalable ETLs in OSCs.

## Supplementary Information

Below is the link to the electronic supplementary material.

Supplementary file1 (DOCX 8394 kb)

## References

[1] L. Feng, Y. Xiang, Z. Li, Q. Li, H. Dong et al., Non-ionic perylene-diimide polymer as universal cathode interlayer for conventional, inverted, and blade-coated organic solar cells. Angew. Chem. Int. Ed. **63**(43), e202410857 (2024). 10.1002/anie.202410857 10.1002/anie.202410857 39073201

[2] Y. Li, M. Han, W. Yang, J. Guo, K. Chang et al., Perylene diimide-based cathode interfacial materials: adjustable molecular structures and conformation, optimized film morphology, and much improved performance of non-fullerene polymer solar cells. Mater. Chem. Front. **3**(9), 1840–1848 (2019). 10.1039/c9qm00236g

[3] Y. Wang, X. Jiang, H. Song, N. Wei, Y. Wang et al., Thickness-insensitive, cyano-modified perylene diimide derivative as a cathode interlayer material for high-efficiency organic solar cells. Acta Phys. Chim. Sin. **41**(3), 100027 (2025). 10.3866/PKU.WHXB202406007

[4] Y. Wang, J. Wen, Z. Shang, Y. Zhong, H. Zhang et al., Enhancing the built-in electric field of thickness-insensitive small molecule cathode interlayers for high-efficiency and stable organic solar cells. Angew. Chem. Int. Ed. **64**(31), e202506252 (2025). 10.1002/anie.202506252 10.1002/anie.202506252 40293795

[5] Y. Wang, J. Wen, Y. Zhong, L. Fu, Z. You et al., Homology-guided zwitterionic interlayers for 21% efficiency non-fullerene organic solar cells. Adv. Mater. **38**(10), e20669 (2026). 10.1002/adma.202520669 41452168 10.1002/adma.202520669

[6] Z. Wang, H. Wang, T. Dai, M. Du, Q. Guo et al., Bay position substituted perylene diimide derivatives as cathode interface materials for high-efficient nonfullerene and fullerene organic photovoltaics. ACS Appl. Energy Mater. **5**(5), 6423–6431 (2022). 10.1021/acsaem.2c00915

[7] J. Yao, B. Qiu, Z.-G. Zhang, L. Xue, R. Wang et al., Cathode engineering with perylene-diimide interlayer enabling over 17% efficiency single-junction organic solar cells. Nat. Commun. **11**, 2726 (2020). 10.1038/s41467-020-16509-w 32483159 10.1038/s41467-020-16509-w PMC7264349

[8] Z. You, J. Wen, W. Liu, Z. Fink, X. Wu et al., Transparent and conductive polyimide-ionene hybrid interlayers for high performance and cost-effective semitransparent organic solar cells. Adv. Mater. **37**(15), 2500450 (2025). 10.1002/adma.202500450 10.1002/adma.202500450 40033990

[9] H. Zhang, W. Han, L. Fu, J. Wen, X. Chang et al., Rationally tailoring simple non-fused ring electron acceptors toward zwitterionic interlayers for organic photovoltaics: the effect of synergetic sidechains engineering. Angew. Chem. Int. Ed. **138**(6), e25358 (2026). 10.1002/ange.202525358 10.1002/anie.202525358 41423833

[10] X. Zhang, Q. Tang, X. Yao, Q. Chen, Z.-G. Zhang et al., Optimizing interface properties of perylene-diimide-based cathode interlayer material by reducing 2-hydroxyethyl groups to achieve organic solar cells with efficiency over 19 %. Nano Energy **137**, 110799 (2025). 10.1016/j.nanoen.2025.110799

[11] Z.-G. Zhang, B. Qi, Z. Jin, D. Chi, Z. Qi et al., Perylene diimides: a thickness-insensitive cathode interlayer for high performance polymer solar cells. Energy Environ. Sci. **7**(6), 1966 (2014). 10.1039/c4ee00022f

[12] D. Zhou, Y. Wang, Y. Li, L. Han, F. Wang et al., N-type small molecule electrolyte cathode interface layer with thickness insensitivity for organic solar cells. Nano Energy **128**, 109890 (2024). 10.1016/j.nanoen.2024.109890

[13] W.T. Hadmojo, F.H. Isikgor, Y. Lin, Z. Ling, Q. He et al., Stable organic solar cells enabled by simultaneous hole and electron interlayer engineering. Energy Environ. Mater. **7**(5), e12712 (2024). 10.1002/eem2.12712

[14] S. Lan, D. Zhou, L. Hu, H. Li, Y. Pu et al., Subtly modulating bay sites of perylene diimide cathode interface layer for high-performance and high-stability non-fullerene organic solar cells. Adv. Funct. Mater. **35**(19), 2419205 (2025). 10.1002/adfm.202419205

[15] Y. Li, B. Huang, X. Zhang, J. Ding, Y. Zhang et al., Lifetime over 10000 hours for organic solar cells with Ir/IrO_*x*_ electron-transporting layer. Nat. Commun. **14**(1), 1–10 (2023). 10.1038/s41467-023-36937-8 36871022 10.1038/s41467-023-36937-8 PMC9985642

[16] T. Le Huyen Mai, S. Jeong, S. Kim, S. Jung, J. Oh et al., Over 18.2%-efficiency organic solar cells with exceptional device stability enabled by bay-area benzamide-functionalized perylene diimide interlayer. Adv. Funct. Mater. **33**(36), 2303386 (2023). 10.1002/adfm.202303386

[17] Z. Yin, S. Mei, P. Gu, H.-Q. Wang, W. Song, Efficient organic solar cells with superior stability based on PM6: BTP-eC9 blend and AZO/Al cathode. iScience **24**(9), 103027 (2021). 10.1016/j.isci.2021.103027 34522867 10.1016/j.isci.2021.103027 PMC8426262

[18] K. Zhong, J. Deng, R. Zeng, J. Xu, Z. Yuan et al., Brominated perylene diimides as cathode interface materials for high efficiency organic solar cells. Adv. Funct. Mater. **35**(6), 2414822 (2025). 10.1002/adfm.202414822

[19] D. Zhou, Y. Pu, Y. Wang, L. Hu, J. Quan et al., Perylene diimide cathode interface layer with siloxane bay-modification for efficient and stable organic solar cells. Chem. Eng. J. **509**, 161155 (2025). 10.1016/j.cej.2025.161155

[20] Q. Liu, Y. Jiang, K. Jin, J. Qin, J. Xu et al., 18% efficiency organic solar cells. Sci. Bull. **65**(4), 272–275 (2020). 10.1016/j.scib.2020.01.001 10.1016/j.scib.2020.01.001 36659090

[21] J. Wen, R. Lin, Y. Wu, H.-C. Hu, Z. Liu et al., Qualified interlayer modifier for organic solar cells with optimized interfacial topography and boosted efficiency based on biomass-derived acid. Chem. Eng. J. **450**, 138169 (2022). 10.1016/j.cej.2022.138169

[22] H. Wu, T. Li, G. Song, Z. He, Y. Cao, Revealing the AcOH-induced dissolution mechanism of alcohol-soluble interlayers. J. Phys. Chem. Lett. **15**(18), 5000–5007 (2024). 10.1021/acs.jpclett.4c00841 38695747 10.1021/acs.jpclett.4c00841

[23] Y. Kim, Z. Sun, T. Le Huyen Mai, J. Park, B. Lee et al., Acceptor-induced reorientation enables vertically and laterally optimized charge-transport devices with a single polymer. Sci. China Chem. (2026). 10.1007/s11426-025-3253-6

[24] C. Li, H. Wonneberger, Perylene imides for organic photovoltaics: yesterday, today, and tomorrow. Adv. Mater. **24**(5), 613–636 (2012). 10.1002/adma.201104447 22228467 10.1002/adma.201104447

[25] A. Liang, Y. Sun, S. Chung, J. Shin, K. Sun et al., Dual-donor-induced crystallinity modulation enables 19.23% efficiency organic solar cells. Nano-Micro Lett. **17**(1), 72 (2024). 10.1007/s40820-024-01576-1 10.1007/s40820-024-01576-1 PMC11602937 39602047

[26] M. Liu, Y. Jiang, D. Liu, J. Wang, Z. Ren et al., Imidazole-functionalized imide interlayers for high performance organic solar cells. ACS Energy Lett. **6**(9), 3228–3235 (2021). 10.1021/acsenergylett.1c01482

[27] D. Li, N. Wei, Y.-N. Chen, X. Wang, X. Han et al., Asymmetric side-group engineering of nonfused ring electron acceptors for high-efficiency thick-film organic solar cells. Nano-Micro Lett. **18**(1), 81 (2025). 10.1007/s40820-025-01905-y 10.1007/s40820-025-01905-y PMC12597859 41207984

[28] T. Le Huyen Mai, Z. Sun, S. Kim, S. Jeong, S. Lee et al., Open-air, green-solvent processed organic solar cells with efficiency approaching 18% and exceptional stability. Energy Environ. Sci. **17**(19), 7435–7444 (2024). 10.1039/d4ee01944j

[29] J. Wen, R. Lin, S. Wang, H.-C. Hu, H. Zhou et al., Semicrystalline cathode interlayer based on morphology control additives using nonconjugated small-molecule zwitterions for efficient organic solar cells. Sol. RRL **6**(11), 2200549 (2022). 10.1002/solr.202200549

[30] T. Lu, F. Chen, Multiwfn: a multifunctional wavefunction analyzer. J. Comput. Chem. **33**(5), 580–592 (2012). 10.1002/jcc.22885 22162017 10.1002/jcc.22885

[31] J.-H. Kim, A. Nizami, Y. Hwangbo, B. Jang, H.-J. Lee et al., Tensile testing of ultra-thin films on water surface. Nat. Commun. **4**, 2520 (2013). 10.1038/ncomms3520 24084684 10.1038/ncomms3520

[32] Z. Wu, C. Sun, S. Dong, X.-F. Jiang, S. Wu et al., N-type water/alcohol-soluble naphthalene diimide-based conjugated polymers for high-performance polymer solar cells. J. Am. Chem. Soc. **138**(6), 2004–2013 (2016). 10.1021/jacs.5b12664 26794827 10.1021/jacs.5b12664

[33] C.G. Tang, M.C.Y. Ang, K.-K. Choo, V. Keerthi, J.-K. Tan et al., Doped polymer semiconductors with ultrahigh and ultralow work functions for ohmic contacts. Nature **539**(7630), 536–540 (2016). 10.1038/nature20133 27882976 10.1038/nature20133

[34] A.K.K. Kyaw, D.H. Wang, V. Gupta, W.L. Leong, L. Ke et al., Intensity dependence of current–voltage characteristics and recombination in high-efficiency solution-processed small-molecule solar cells. ACS Nano **7**(5), 4569–4577 (2013). 10.1021/nn401267s 23597037 10.1021/nn401267s

[35] Z. Sun, H. Ma, T. Le Huyen Mai, J. Park, S. Jeong et al., Nanoscale balance of energy loss and quantum efficiency for high-efficiency polythiophene-based organic solar cells. ACS Nano **19**(1), 1026–1035 (2025). 10.1021/acsnano.4c12705 39722448 10.1021/acsnano.4c12705

[36] Z. Sun, H. Ma, S. Yang, Y. Cho, S. Lee et al., Insight into designing high-performance polythiophenes for reduced urbach energy and nonradiative recombination in organic solar cells. Adv. Funct. Mater. **34**(39), 2403093 (2024). 10.1002/adfm.202403093

[37] Y. Wang, C. Gao, W. Lei, T. Yang, Z. Liang et al., Achieving 20% toluene-processed binary organic solar cells *via* secondary regulation of donor aggregation in sequential processing. Nano-Micro Lett. **17**(1), 206 (2025). 10.1007/s40820-025-01715-2 10.1007/s40820-025-01715-2 PMC11961838 40167593

[38] T. Lu, F. Chen, Bond order analysis based on the Laplacian of electron density in fuzzy overlap space. J. Phys. Chem. A **117**(14), 3100–3108 (2013). 10.1021/jp4010345 23514314 10.1021/jp4010345

[39] P. Bilski, K. Reszka, M. Bilska, C.F. Chignell, Oxidation of the spin trap 5, 5-dimethyl-1-pyrroline N-oxide by singlet oxygen in aqueous solution. J. Am. Chem. Soc. **118**(6), 1330–1338 (1996). 10.1021/ja952140s

[40] E. Braxton, D.J. Fox, B.G. Breeze, J.J. Tully, K.J. Levey et al., Electron paramagnetic resonance for the detection of electrochemically generated hydroxyl radicals: issues associated with electrochemical oxidation of the spin trap. ACS Meas. Sci. Au **3**(1), 21–31 (2023). 10.1021/acsmeasuresciau.2c00049 36817006 10.1021/acsmeasuresciau.2c00049 PMC9936800

[41] J.-L. Clément, N. Ferré, D. Siri, H. Karoui, A. Rockenbauer et al., Assignment of the EPR spectrum of 5, 5-dimethyl-1-pyrroline *N*-oxide (DMPO) superoxide spin adduct. J. Org. Chem. **70**(4), 1198–1203 (2005). 10.1021/jo048518z 15704951 10.1021/jo048518z

[42] K.U. Ingold, D.A. Pratt, Advances in radical-trapping antioxidant chemistry in the 21st century: a kinetics and mechanisms perspective. Chem. Rev. **114**(18), 9022–9046 (2014). 10.1021/cr500226n 25180889 10.1021/cr500226n

[43] H.T. Nicolai, M. Kuik, G.A.H. Wetzelaer, B. de Boer, C. Campbell et al., Unification of trap-limited electron transport in semiconducting polymers. Nat. Mater. **11**(10), 882–887 (2012). 10.1038/nmat3384 22842510 10.1038/nmat3384

[44] M. Salvador, N. Gasparini, J.D. Perea, S.H. Paleti, A. Distler et al., Suppressing photooxidation of conjugated polymers and their blends with fullerenes through nickel chelates. Energy Environ. Sci. **10**(9), 2005–2016 (2017). 10.1039/c7ee01403a

[45] H.-I. Un, Y.-Q. Zheng, K. Shi, J.-Y. Wang, J. Pei, Air- and active hydrogen-induced electron trapping and operational instability in n-type polymer field-effect transistors. Adv. Funct. Mater. **27**(11), 1605058 (2017). 10.1002/adfm.201605058

[46] O.R. Yamilova, I.V. Martynov, A.S. Brandvold, I.V. Klimovich, A.H. Balzer et al., What is killing organic photovoltaics: light-induced crosslinking as a general degradation pathway of organic conjugated molecules. Adv. Energy Mater. **10**(7), 1903163 (2020). 10.1002/aenm.201903163

[47] U. Zschieschang, K. Amsharov, M. Jansen, K. Kern, H. Klauk et al., Separating the impact of oxygen and water on the long-term stability of n-channel perylene diimide thin-film transistors. Org. Electron. **26**, 340–344 (2015). 10.1016/j.orgel.2015.07.060

[48] G. Zuo, M. Linares, T. Upreti, M. Kemerink, General rule for the energy of water-induced traps in organic semiconductors. Nat. Mater. **18**(6), 588–593 (2019). 10.1038/s41563-019-0347-y 31011215 10.1038/s41563-019-0347-y

[49] D.E. Shen, A.W. Lang, G.S. Collier, A.M. Österholm, E.M. Smith et al., Enhancement of photostability through side chain tuning in dioxythiophene-based conjugated polymers. Chem. Mater. **34**(3), 1041–1051 (2022). 10.1021/acs.chemmater.1c03317

[50] R.A. Street, Y. Yang, B.C. Thompson, I. McCulloch, Capacitance spectroscopy of light induced trap states in organic solar cells. J. Phys. Chem. C **120**(39), 22169–22178 (2016). 10.1021/acs.jpcc.6b06561

[51] G. Garcia-Belmonte, P.P. Boix, J. Bisquert, M. Sessolo, H.J. Bolink, Simultaneous determination of carrier lifetime and electron density-of-states in P3HT: PCBM organic solar cells under illumination by impedance spectroscopy. Sol. Energy Mater. Sol. Cells **94**(2), 366–375 (2010). 10.1016/j.solmat.2009.10.015

[52] D. Cao, L. Zhong, Z. Sun, J. Sun, H.T. Le Mai et al., Allylrhodanine-processed all-small-molecule organic solar cell achieves an 18.43% efficiency breakthrough. Nat. Commun. **17**, 2105 (2026). 10.1038/s41467-026-68924-0 41605949 10.1038/s41467-026-68924-0 PMC12953913

[53] S. Jeong, Z. Sun, Y. Cho, S. Yang, T. Le Huyen Mai et al., 3D conjugated nonflat biphenyl side chains: their exclusive role in inducing negative electrostatic potential in efficient organic solar cells. Small **21**(41), e09667 (2025). 10.1002/smll.202509667 40878401 10.1002/smll.202509667 PMC12530028

[54] Z. Li, F. Gao, N.C. Greenham, C.R. McNeill, Comparison of the operation of polymer/fullerene, polymer/polymer, and polymer/nanocrystal solar cells: a transient photocurrent and photovoltage study. Adv. Funct. Mater. **21**(8), 1419–1431 (2011). 10.1002/adfm.201002154

[55] J.-S. Kim, J.-H. Kim, W. Lee, H. Yu, H.J. Kim et al., Tuning mechanical and optoelectrical properties of poly(3-hexylthiophene) through systematic regioregularity control. Macromolecules **48**(13), 4339–4346 (2015). 10.1021/acs.macromol.5b00524

[56] W. Kim, J. Choi, J.-H. Kim, T. Kim, C. Lee et al., Comparative study of the mechanical properties of all-polymer and fullerene–polymer solar cells: the importance of polymer acceptors for high fracture resistance. Chem. Mater. **30**(6), 2102–2111 (2018). 10.1021/acs.chemmater.8b00172

[57] X. Yang, P. Gao, Z. Yang, J. Zhu, F. Huang et al., Optimizing ultrathin Ag films for high performance oxide-metal-oxide flexible transparent electrodes through surface energy modulation and template-stripping procedures. Sci. Rep. **7**, 44576 (2017). 10.1038/srep44576 28291229 10.1038/srep44576 PMC5349598

