# Supplementary material for: Polar-Twisted Electron-Transport Layer Simultaneously Breaks Efficiency-Flexibility-Cost Limits in Organic Solar Cells
Source: Nanomicro Lett. 2026 Aug 13;19:33. doi: 10.1007/s40820-026-02330-5 (PMC13473058; doi:10.1007/s40820-026-02330-5)
Supplement: Supplementary file 1 [file 40820_2026_2330_MOESM1_ESM.docx]

Supporting Information for

**Polar-Twisted Electron-Transport Layer Simultaneously Breaks Efficiency-Flexibility-Cost Limits in Organic Solar Cells**

Thi Le Huyen Mai^1^, Zhe Sun^1^*, Won Jung Kang^2^, Seunglok Lee^1^, Taek-Soo Kim^2^, and Changduk Yang^1,3,4^*

^1^Department of Energy Engineering, School of Energy and Chemical Engineering, Ulsan National Institute of Science and Technology (UNIST), 50 UNIST-gil, Ulju-gun, Ulsan 44919, Republic of Korea

^2^ Department of Mechanical Engineering, Korea Advanced Institute of Science and Technology (KAIST), 291 Daehak-ro, Yuseong-gu, Daejeon, 34141, Republic of Korea

^3^Graduate School of Carbon Neutrality, Ulsan National Institute of Science and Technology (UNIST), 50 UNIST-gil, Ulju-gun, Ulsan 44919, Republic of Korea

^4^UNIST InnoCORE AI-Space Solar Initiative, Ulsan National Institute of Science and Technology (UNIST), Ulsan, 44919, Republic of Korea

*Corresponding authors. E-mail: [yang@unist.ac.kr](mailto:yang@unist.ac.kr) (Changduk Yang); [zhesun@unist.ac.kr](mailto:zhesun@unist.ac.kr) (Zhe Sun)

**Supporting Experiments**

*Synthesis of 6-bromo-N,N-dimethyl-2-naphthamide (1):* A solution of 6-bromo-2-naphthoic acid (5.0 g, 19.91 mmol) in dichloromethane (40 mL) was prepared and stirred at ambient temperature. Oxalyl chloride (2.80 mL, 31.86 mmol) was added carefully, followed by a catalytic trace of DMF, and the mixture was allowed to stir for 2 h. The reaction was then cooled to 0 °C, after which dimethylamine (25 mL) was introduced dropwise. Stirring was continued overnight at room temperature. The reaction was quenched with water, and the organic layer was separated with dichloromethane, washed successively with 1 M HCl, saturated NaHCO_3_, and brine. The combined organic extracts were dried over MgSO_4_, filtered, and concentrated under reduced pressure. Purification of the residue by flash chromatography on silica gel furnished compound **1** in 84 % yield (4.60 g) as a colourless oil that crystallises to a white solid upon refrigeration. ^1^H NMR (400 MHz, CDCl_3_, δ): 8.02 (s, 1H), 7.88 (s, 1H), 7.77 (dd, *J*= 8.0 Hz, 2H), 7.61 (dd, *J*= 8.0 Hz, 2H), 3.16 (s, 3H), 3.03 (s, 3H).

*Synthesis of N,N-dimethyl-2-naphthamide-6-boronic acid pinacol ester* *(2):* Combine compound **1** (3.0 g, 10.79 mmol), Pd(dppf)Cl_2_.DCM (0.14 g, 0.53 mmol), bis(pinacolato)diboron (4.10 g, 16.17 mmol) and potassium acetate (3.17 g, 32.35 mmol) in 100 mL of 1,4-dioxane. Stir the mixture under argon at 100 °C for 24 h. After it cools to room temperature, extract with ethyl acetate and wash the organic phase successively with water and brine. Dry over anhydrous MgSO_4_, filter, and remove the solvent under reduced pressure. Purify the residue by silica-gel column chromatography (ethyl acetate : hexane) to obtain compound **2** as a white solid (3.20 g, 91 % yield). ^1^H NMR (400 MHz, CDCl_3_, δ): 8.37 (s, 1H), 7.88 (m, 4H), 7.51 (d, *J*= 7.8 Hz, 1H), 3.16 (s, 3H), 3.02 (s, 3H), 1.39 (s, 12H).

*Synthesis of PDIN-NAP*: PDIN-Br_2_ (0.20 g, 0.27 mmol), naphthalene-2-boronic acid (0.14 g, 0.84 mmol), potassium carbonate (0.77 g, 5.56 mmol) and Pd(pph_3_)_4_ (0.016 g, 0.013 mmol) was dissolved in 24 mL/12 mL/6 mL toluene/ethanol/water. Then, the mixture reaction was stirred at 100 °C for 24 h under the argon atmosphere. After cooling down to room temperature, the crude mixture was extracted with chloroform, washed with water, brine. Next, solvents were evaporated under reduced pressure after the combined organic layer was dried with anhydrous MgSO_4_. The residue was purified by column chromatography on silica gel (chloroform : triethylamine : methanol) to obtain **PDIN-NAP** as the red solid (0.19 g, 82%). ^1^H NMR (400 MHz, CDCl_3_, δ): 8.74 (s, 2H), 7.92 (m, 14H), 7.61 (m, 4H), 4.25 (m, 4H), 2.45 (m, 4H), 2.26 (s, 12H), 1.93 (m, 4H). ^13^C NMR (151 MHz, CDCl_3_, δ): 163.33, 140.84, 139.40, 135.59, 134.60, 133.01, 132.39, 130.38, 129.70, 129.39, 129.10, 128.44, 128.27, 127.94, 127.47, 127.15, 126.96, 126.71, 122.28, 121.83, 57.20, 45.33, 38.96, 26.04. HRMS (MALDI-TOF): calcd for C_54_H_44_N_4_O_4_, 812.97. Found: 813.24.

*Synthesis of H75-DMA*: Synthesized according to a similar procedure to PDIN-NAP by utilizing PDIN-Br_2_ (0.10 g, 0.14 mmol), **2** (0.14 g, 0.42 mmol), potassium carbonate (0.38 g, 2.78 mmol) and Pd(pph_3_)_4_ (0.008 g, 0.006 mmol) and toluene/ethanol/water (18 mL/9 mL/4.5 mL). **H75-DMA** as dark red solid (0.11 g, 85% yield). ^1^H NMR (400 MHz, CDCl_3_, δ): 8.75 (s, 2H), 7.99 (m, 14H), 7.63 (m, 2H), 4.24 (m, 4H), 3.22 (d, 12H), 2.44 (m, 4H), 2.26 (s, 12H), 1.92 (m, 4H). ^13^C NMR (151 MHz, CDCl_3_, δ): 171.21, 163.29, 140.52, 135.50, 134.95, 134.53, 132.47, 132.30, 130.46, 130.20, 129.50, 129.12, 128.69, 128.11, 127.60, 127.57, 126.76, 125.60, 122.42, 121.98, 57.18, 45.35, 39.79, 38.98, 35.50, 26.08. HRMS (MALDI-TOF): calcd for C_60_H_54_N_6_O_6_, 954.41. Found: 955.54.

**Supplementary Figures and Tables**

**Table S1** Optical and electrochemical properties of PDIN, PDIN-NAP, and H75-DMA

[a] Data collected both from the samples dissolved in methanol and from their corresponding thin films cast onto glass substrates. [b] Obtained from the onset wavelength of the UV–vis absorption spectra of the thin films. [c] HOMO and LUMO energies were derived from the initial oxidation and reduction potentials using E_HOMO_ = −(E_ox_^onset^ − E_Fc_^onset^ + 4.8) eV, E_LUMO_ = −(E_red_^onset^ − E_Fc_^onset^ + 4.8 ) eV; the electrochemical bandgap E_g_^CV^ was then calculated as E_HOMO_ – E_LUMO._

**Table S2** The work function and the injection barriers for hole of ETLs

**Table S3** The electron mobility and conductivity of ETLs

**Table S4** Normalized photovoltaic parameters (%) of devices based on PDIN, PDINN, PDIN-NAP, and H75-DMA as the ETL on D18-Cl:N3 under ambient environment stability (40-60% RH, 25 ℃, unencapsulated)

[a] the average PCE values are obtained from at least 8 cells.

**Table S5** Summary of XPS fitting parameters for the C 1*s* and N 1*s* spectra of fresh and aged ETL films

**Table S6** The exciton dissociation probability and charge collection probability values of fresh and aged devices with PDIN, PDINN, PDIN-NAP, and H75-DMA ETLs

**Table S7** The TPV and TPC lifetime values of fresh and aged devices with PDIN, PDINN, PDIN-NAP, and H75-DMA ETLs.

[a] the average values are obtained from 3 cells.

**Table S8** Normalized photovoltaic parameters of devices based on PDIN, PDIN-NAP, and H75-DMA as the ETL on D18-Cl:N3 under bending cycles

[a] the average PCE values are obtained from at least 6 cells.

**Table S9** Surface-energy components calculated from three-liquid contact-angle measurements using the Owens–Wendt method

**Table S10** Summary of PCE, AVT and LUE values for comparative literature data in Fig. 6h

**Fig. S1** The synthetic route of PDIN-NAP and H75-DMA

**Fig. S2** ^1^H NMR spectrum of 6-bromo-N,N-dimethyl-2-naphthamide

**Fig. S3** ^1^H NMR spectrum of *N,N*-dimethyl-2-naphthamide-6-boronic acid pinacol ester

**Fig. S4** ^1^H NMR spectrum of PDIN-NAP

**Fig. S5** ^1^H NMR spectrum of H75-DMA

**Fig. S6** MALDI-TOF mass spectrometry of PDIN-NAP

**Fig. S7** MALDI-TOF mass spectrometry of H75-DMA

**Fig. S8** The B3LYP/6-31G levels optimize geometries. Electrostatic potential (ESP) distribution and dipole moment for PDIN (a), PDIN-NAP (b), and H75-DMA (c). The front and side views of (d, g) PDIN, (e, h) PDIN-NAP, and (f, i) H75-DMA

**Fig. S9** Normalized UV–vis absorption spectra of PDIN, PDIN-NAP, and H75-DMA in methanol solution and film states

**Fig. S10** Schematic diagram of energy level and the cyclic voltammetry of PDIN, PDIN-NAP, and H75-DMA

**Fig. S11** UPS spectra of without and with PDIN, PDIN-NAP, and H75-DMA ETL-deposited on Ag electrode

**Fig. S12** Electron spin resonance spectra of PDIN, PDIN-NAP, and H75-DMA in solid

**Fig. S13** SCLC plots of electron only devices with the configuration of ITO/ZnO/ETL/Ag

**Fig. S14** The *J-V* curve of devices with configuration of ITO/ETL/Ag

**Fig. S15** The AFM height images of without and with PDIN, PDIN-NAP, and H75-DMA ETL-deposited on active layer (AL) films

**Fig. S16** Illustration of the morphology transition from the highly crystalline, edge-on orientation of PDIN to the amorphous-like orientation, low-crystallinity texture achieved with H75-DMA

**Fig. S17** Chemical structures of the ETL, HTL and active layer materials used in this work

**Fig. S18** a) Light intensity (*I*_light_) dependence of *V*_OC_ and J_SC_ plots of the optimized device with PDIN, PDIN-Naph and H75-NB as ETLs based on D18-Cl:N3 system. Note: *V*_OC_ versus *I*_light_ can be described by the equation of *V*_OC_ ∝ n (kT/q) ln(*I*_light_), where k, T, and q denote the Boltzmann constant, Kelvin temperature, and elementary charge, respectively. b**)** J*_S_*_C_ versus *I*_light_ can be described by the equation of J_SC_ ∝ *I*^α^_light_, where α should be equal to 1 if the bimolecular recombination of the device is negligible

**Fig. S19**  **a** The *J*-*V* curves of the optimized devices based on PM6:L8-BO with H75-DMA ETLs under illumination of AM 1.5 G, 100 mW cm^−2^. **b** The EQE spectra and integrated current densities of the representative devices

**Fig. S20** The normalized stability test **a** *V*_OC_ , **b** FF, and **c** *J*_SC_ of the unencapsulated devices with PDIN, PDINN, PDIN-NAP and H75-DMA under 40-60% RH environment at 25 °C.

**Fig. S21** Normalized PCE evolution of unencapsulated OSCs employing different ETLs under continuous 1 sun illumination at 55 °C and 40–60% RH.

**Fig. S22** Dark thermal aging stability of OSCs under N_2_ at 85 °C.

**Fig. S23** LBO values for C-H bonds, C-C bonds, and C-N bonds of PDIN, PDINN, PDIN-NAP, and H75-DMA

**Fig. S24** a Schematic diagram of the C–H/N-H bonds in the ETLs (secondary amine: PDINN, tertiary amine: PDIN, PDIN-NAP, and 75-DMA) for calculating LBO values. b Laplacian bond order values for the hydrogen abstraction of amine side-chains

**Fig. S25** Laplacian bond order values for C-H/C-C/C-N of PDIN after protonation by acid

**Fig. S26** Laplacian bond order values for C-C/C-H belong to the PDI backbone

**Fig. S27** The ^1^H NMR spectra of fresh PDIN, PDINN, PDIN-NAP, and H75-DMA solutions in methanol-d_4_, as well as those aged under ambient conditions (before and after one week (168 hours) at 25 °C and 40-60% relative humidity)

**Fig. S28** The ESR signals of OH^•^ (DMPO–^•^OH) for fresh and aged samples under ambient conditions over 168 hours of PDIN, PDINN, PDIN-NAP, and H75-DMA in water

**Fig. S29** The FT-IR spectra from fresh and aged PDIN, PDINN, PDIN-NAP, and H75-DMA thin films

**Fig. S30** a Reactive Oxygen-Species (ROS) generation pathways in organic semiconductors. b Acid-assisted methanol solubility of PDI-based interlayers. c Protonation- and ROS-Driven Degradation Routes of PDIN and PDINN

**Fig. S31** The Nyquist plot from fresh and aged PDIN, PDINN, PDIN-NAP, and H75-DMA thin films

**Fig. S32** Optical microscope images of active layer (AL)/PDIN, AL/PDIN-NAP, and AL/H75-DMA films obtained during pseudo-free-standing tensile testing

**Fig. S33** Toughness of PDIN, PDIN-NAP and H75-DMA on active layer

**Fig. S34** Crack-onset strain identification for PDIN, PDIN-NAP, and H75-DMA on the active layer

**Fig. S35** Cohesive fracture energy (G_c_) of PDIN, PDIN-NAP and H75-DMA on active layer

**Fig. S36** The EQE spectra and integrated current densities of the best flexible devices utilizing PDIN, PDIN-NAP, and H75-DMA ETLs.

**Fig. S37** PCE**,** FF, *V*_OC_, and *J*_SC_ histograms of the flexible devices utilizing PDIN, PDIN-NAP, and H75-DMA ETLs.

**Fig. S38** a Schematic of Ag thin-film nucleation and growth on ETL surfaces, illustrating layer-by-layer (Frank–van der Merwe), island (Volmer–Weber), and layer-plus-island (Stranski–Krastanov) modes. b Sheet resistance of 20 nm Ag films deposited on the ETLs

**Fig. S39** Contact-angle images of three probe liquids for surface-energy analysis. From top to bottom: deionized water (DI), ethylene glycol (EG), and diiodomethane (DIM) droplets on ITO/HTL/BHJ/PDIN, ITO/HTL/BHJ/PDIN-NAP, and ITO/HTL/BHJ/H75-DMA substrates

**Fig. S40** Indicative market prices: Ag vs Cu and the *J*-*V* curves of the optimized devices utilizing a Cu electrode with PDIN, PDIN-NAP, and H75-DMA ETLs in the D18-Cl:N3 system under AM 1.5 G illumination at 100 mW cm^−2^

**Fig. S41** ^1^H NMR integrated profiles of fresh and aged PDIN in methanol–d_4_

**Fig. S42** ^1^H NMR integrated profiles of fresh and aged PDINN in methanol–d_4_

**Fig. S43** ^1^H NMR integrated profiles of fresh and aged PDIN-NAP in methanol–d_4_

**Fig. S44** ^1^H NMR integrated profiles of fresh and aged H75-DMA in methanol–d_4_

**Fig. S45** ^13^C NMR spectrum of PDIN-NAP

**Fig. S46** ^13^C NMR spectrum of H75-DMA

**Supplementary References**

[S1] W. Jose da Silva, H.P. Kim, A. Rashid bin Mohd Yusoff, J. Jang, Transparent flexible organic solar cells with 6.87% efficiency manufactured by an all-solution process. Nanoscale **5**(19), 9324 (2013). <https://doi.org/10.1039/c3nr03011c>
[S2] J. Czolk, D. Landerer, M. Koppitz, D. Nass, A. Colsmann, Highly efficient, mechanically flexible, semi-transparent organic solar cells doctor bladed from non-halogenated solvents. Adv. Mater. Technol. **1**(9), 1600184 (2016). <https://doi.org/10.1002/admt.201600184>
[S3] Y. Liu, P. Cheng, T. Li, R. Wang, Y. Li et al., Unraveling sunlight by transparent organic semiconductors toward photovoltaic and photosynthesis. ACS Nano **13**(2), 1071–1077 (2019). <https://doi.org/10.1021/acsnano.8b08577>
[S4] J. Zhang, G. Xu, F. Tao, G. Zeng, M. Zhang et al., Highly efficient semitransparent organic solar cells with color rendering index approaching 100. Adv. Mater. **31**(10), 1807159 (2019). <https://doi.org/10.1002/adma.201807159>
[S5] W. Zheng, X. Luo, Y. Zhang, C. Ye, A. Qin et al., Efficient low-cost all-flexible microcavity semitransparent polymer solar cells enabled by polymer flexible one-dimensional photonic crystals. ACS Appl. Mater. Interfaces **12**(20), 23190–23198 (2020). <https://doi.org/10.1021/acsami.0c03508>
[S6] W. Song, B. Fanady, R. Peng, L. Hong, L. Wu et al., Foldable semitransparent organic solar cells for photovoltaic and photosynthesis. Adv. Energy Mater. **10**(15), 2000136 (2020). <https://doi.org/10.1002/aenm.202000136>
[S7] Y. Wang, G. Wang, Y. Xing, M.A. Adil, W. Ali Memon et al., Top and bottom electrode optimization enabled high-performance flexible and semi-transparent organic solar cells. Mater. Chem. Front. **5**(11), 4310–4316 (2021). <https://doi.org/10.1039/d1qm00151e>
[S8] H. Lee, S. Jeong, J.-H. Kim, Y.-R. Jo, H.J. Eun et al., Ultra-flexible semitransparent organic photovoltaics. npj Flex. Electron. **7**, 27 (2023). <https://doi.org/10.1038/s41528-023-00260-5>
[S9] S. Sun, W. Zha, C. Tian, Z. Wei, Q. Luo et al., Solution processed semi-transparent organic solar cells over 50% visible transmittance enabled by silver nanowire electrode with sandwich structure. Adv. Mater. **35**(46), 2305092 (2023). <https://doi.org/10.1002/adma.202305092>
[S10] H. Liu, X. Yi, J. Wu, Y. Li, H. Tang et al., All-solution-processed flexible semitransparent organic solar cells based on sprayed silver nanowire composites as top electrodes. Mater. Sci. Semicond. Process. **173**, 108113 (2024). <https://doi.org/10.1016/j.mssp.2024.108113>
[S11] Y. Liao, K. Xie, J. Wu, N. Zhao, Y. Lin et al., Enhancing the durability and efficiency of flexible semi-transparent organic solar cells with silver mesh electrode. IEEE Trans. Electron Devices **72**(6), 3099–3105 (2025). <https://doi.org/10.1109/ted.2025.3559887>
[S12] B. Shi, J. Wang, X. Bao, W. Ma, H. Yan, One plastic improves the shortages of photovoltaic and mechanical properties in flexible semitransparent organic photovoltaics. Chem. Commun. **61**(100), 19861–19864 (2025). <https://doi.org/10.1039/d5cc05795g>
